# Respiratory self navigated whole-heart angiography with ultra-small super-paramagnetic iron oxide particles: a feasibility study

**DOI:** 10.1186/1532-429X-18-S1-P56

**Published:** 2016-01-27

**Authors:** Davide Piccini, Peter J Weale, Annette S Cooper, Rachael O Forsythe, David Newby, Scott Semple

**Affiliations:** 1 Advanced Clinical Imaging Technology, Siemens Healthcare, Lausanne, Switzerland; 2 Siemens Healthcare UK, Camberley, UK; 3 grid.4305.20000000419367988Clinical Research Imaging Centre, University of Edinburgh, Edinburgh, UK; 4 grid.4305.20000000419367988Centre for Cardiovascular Science, University of Edinburgh, Edinburgh, UK

## Background

Self-navigation (SN) in 3D radial segmented MR imaging [1] has been shown to be an efficient and robust method for depiction of coronary luminal anatomy using MRI. This method has largely been used at 1.5T using SSFP readouts to provide the high blood to myocardium contrast required for vessel segmentation and SN. However, bSSFP imaging is often confounded by off-resonance artifacts at higher field strengths and conventional Gadolinium based contrast agents combined with inversion-recovery preparation and gradient-echo readouts often cannot achieve a constant signal throughout the scan. Variable contrast throughout the acquisition can cause deterioration in the SN signal and therefore of the motion correction. Here we investigate the use of an intravascular contrast agent (USPIO) with long half-life as a potential method to improve the performance of the SN approach at 3T.

## Methods

Eight healthy volunteers were recruited in accordance with local ethical approval. Segmented, 3D ECG triggered, SN data was acquired using a prototype 3D radial sequence [2] on a 3T system (MAGNETOM Verio, Siemens Healthcare). Optimal tissue contrast was empirically obtained using an inversion time of 300 ms to provide good myocardial suppression and appropriate enhancement of the blood pool over at least a 40-50 min window (allowing repeat imaging if required). Intravenous ferumoxytol (510 mg of elemental iron (Fe) in 17 ml (30 mg/ml)) at a dose of 4 mg of iron (Fe)/kg of body weight was administered over a 15 min infusion (in accordance with international guidelines). In all cases both diastolic and systolic imaging was undertaken and evaluated qualitatively for the reliability of the self-navigation signal and the final image quality.

## Results

**Figure 1:**
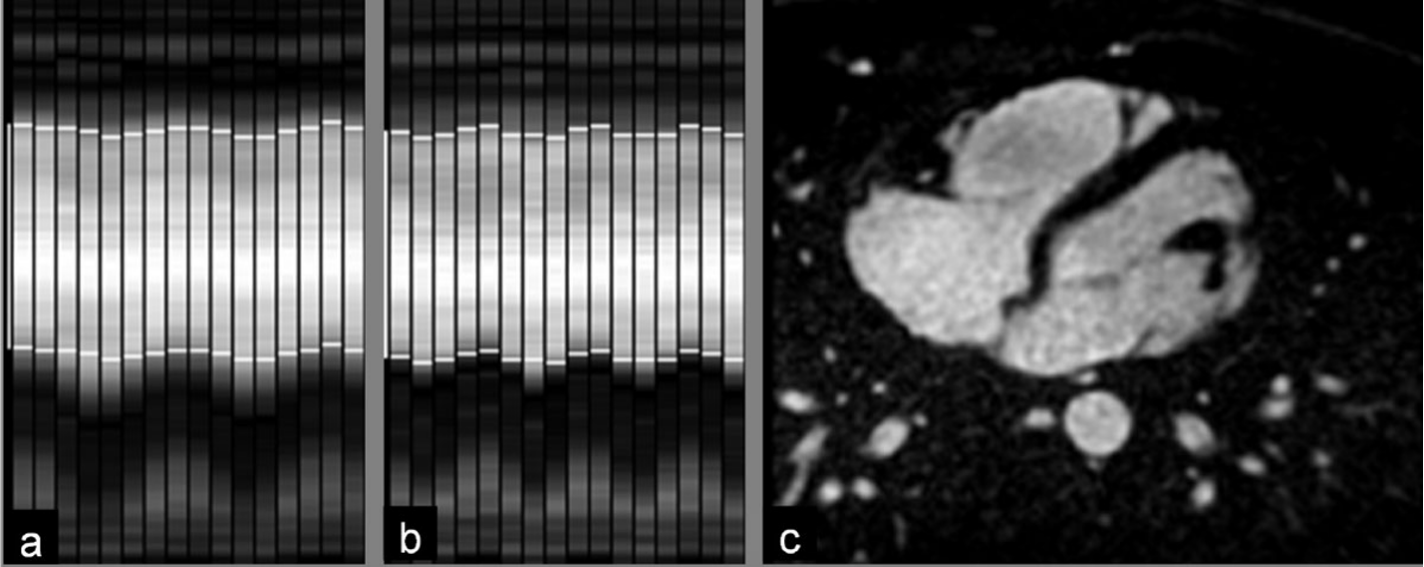
**Examples of self-navigation signal in a diastolic (a) and systolic (b) acquisition, respectively acquired immediately and 10 minutes after injection**. The bright signal from the blood pool is well visible on the dark background in both examples and the tracking (bright horizontal lines) works very reliably. The image in (c) shows an axial reformat that highlights the excellent contrast between blood and background. Both large and smaller vessels appear well defined.

**Figure 2:**
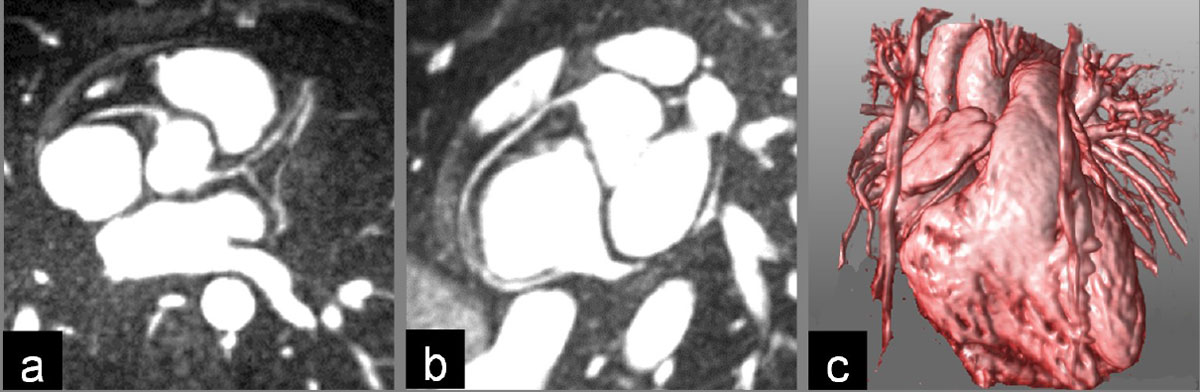
**Example of reformatted coronary arteries and volume rendering for the same volunteer from a diastolic dataset**. (a) Reformat of the origins of all coronary arteries, (b) curved reformat of the right coronary artery, and (c) volume rendering.

In almost all cases both the SN signal and the final image quality were good - with the two confounding issues observed in individual cases, 1) sub-optimal fat saturation reducing CNR in small vessels and 2) large non periodic motion (heavy inspirations) in subjects with less regular respiratory pattern. The long half-life of the contrast agent enabled an identical inversion time to be used for both systolic and diastolic acquisitions, providing good tissue contrast for both datasets (Fig 1, 2).

## Conclusions

We have demonstrated the feasibility of T1 shortening USPIO based contrast enhanced respiratory SN at 3T. The long lasting contrast enhancement provides a temporally and spatially constant signal and enables high quality imaging. Potential improvements include methods for automated rejection of data acquired during anomalous respiratory motion and further optimization of image contrast. The longer acting contrast agent allows additional data to be re-acquired in problematic cases. As USPIOs can be used as a biomarker for inflammation there is the potential to use this method as both an angiographic method and as a method for detection of concentration of USPIO in areas of inflammation.

